# Surgical caps displaying team members' names and roles improve effective communication in the operating room: a pilot study

**DOI:** 10.1186/s13037-021-00301-w

**Published:** 2021-07-28

**Authors:** Ned Douglas, Sophie Demeduik, Kate Conlan, Priscilla Salmon, Brian Chee, Taylor Sullivan, David Heelan, John Ozcan, Gareth Symons, Candida Marane

**Affiliations:** 1 Department of Anaesthesia and Pain Management, The Royal Melbourne Hospital, Melbourne, VIC Australia; 2 Centre for Integrated Critical Care, University of Melbourne, Melbourne, VIC Australia; 3 Department of Midwifery Education, Western Health, St. Albans, VIC Australia; 4 Operating Theatre, Western Health, St. Albans, VIC Australia; 5 Department of Anaesthesia, Pain and Perioperative Medicine, Western Health, Locked Bag 2, Footscray, VIC 3011 Australia

**Keywords:** Patient safety, Teamwork

## Abstract

**Background:**

Teamwork in the operating theatre is a complex emergent phenomenon and is driven by cooperative relationships between staff. A foundational requirement for teamwork is the ability to communicate effectively, and in particular, knowing each other’s name. Many operating theatre staff do not know each other’s name, even after formal team introductions. The use of theatre caps to display a staff member’s name and role has been suggested to improve communication and teamwork.

**Methods:**

We hypothesized that the implementation of scrub hats with individual team members' names and roles would improve the perceived quality and effectiveness of communication in the operating theatre. A pilot project was designed as a pre-/post-implementation questionnaire sent to 236 operating room staff members at a general hospital in suburban Melbourne, Victoria, Australia, between November 6 to December 18, 2018. Participants included medical practitioners (anaesthetists, surgeons, obstetricians and gynaecologists), nurses (anaesthetic, scrub/scout and paediatric nurses), midwives and theatre technicians. The primary outcome was a change in perceived teamwork score, measured using a five position Likert scale.

**Results:**

Of 236 enrolled participants, 107 (45%) completed both the pre and post intervention surveys. The median perceived teamwork response of four did not change after the intervention, though the number of low scores was reduced (*p* = 0.015). In a pre-planned subgroup analysis, the median perceived teamwork score rose for midwives from three to four (*p* < 0.001), while for other craft groups remained similar. The median number of staff members in theatre that a participant did not know the name of reduced from three to two (*p* < 0.001). Participants reported knowing the names of all staff members present in the theatre more frequently after the intervention (31% vs 15%, *p* < 0.001). The reported rate of formal team introductions was not significantly different after the intervention (34.7% vs 47.7% *p* = 0.058).

**Conclusions:**

In this study, we found that wearing caps displaying name and role appeared to improve perceived teamwork and improve communication between staff members working in the operating theatre.

## Introduction

Team members knowing and using each other’s names leads to better communication and is a recognised component of good team function, particularly in the event of clinical crisis [1]. Clinical incidents are common, and often occur due to poor communication between surgical team members [2, 3]. Good team communication during surgery has been linked to improved team function [4]. Directing communication to a specific staff member appears to improve team function [5, 6]. Teams in operating theatres are often large and include people who may have met for the first time at the start of the operating list [7]. Even when staff have worked together previously, the rapid changeover of staff and fact that hundreds of personnel may work in the theatre department means that situations frequently arise in which staff do not know the names of everyone working in the theatre [8–10].

One of the features of highly functioning teams is that team members address each other using their first names, and that tasks are allocated to a specific person, rather than to the generic “someone” in the room [1]. Good team behaviors were associated with greater satisfaction at work and better patient safety outcomes [10]. It is known that surgical team members often do not know the names of people they are working with, particularly if the person rarely attends the operating theatre or is of a lower perceived status, and that this deficit can contribute to team dysfunction [7]. These problems are perceived as reducing effective communication during surgery [11].

Checklists and formal introductions have been advocated to address these problems [12]. While checklists are effective at reducing patient harm [13], the available evidence suggests some staff recalled as few as 30% of other staff’s names after the WHO recommended Team-Time-Out process [14]. More sophisticated techniques to improve name retrieval require significant investment by participants, and this may not be feasible in the context of a busy operating list [15]. Although visible nametags worn on clothing are an effective solution in a ward environment, the need for sterile gowns and equipment make these ineffective in the theatre environment.

In the operating theatre all staff members wear scrub hats to contain their hair and prevent hair and skin cells from contaminating the surgical field. One proposed solution to increase awareness of team members first names was to display staff members name and role on the front surface of their theatre hat. This idea had received significant attention in the media and to a limited degree in anaesthetic [16, 17] and obstetric [18] literature. This idea had not been extensively tested despite having a high degree of face validity, and existing evidence was limited to specific craft groups or operation types. This trial aimed to assess the impact of placing a staff member’s name and job-role on their hat on perceived teamwork performance and satisfaction.

## Methods

The study was approved by the Western Health Human Low Risk Research Panel, a subsidiary panel of the Melbourne Health Human Research Ethics Committee. Written informed consent was obtained from all participants.

The study design was a before-and-after quality improvement trial of scrub hats displaying a staff member’s name and role. A before and after design was chosen because it was not feasible to randomize patient exposure to the intervention within the context of the theatre’s working practices.

The study was conducted at a metropolitan general hospital of 600 beds, located in outer metropolitan Melbourne, Victoria, Australia. The theatre complex incorporated six operating theatres and performed all adult and paediatric general, orthopaedic, vascular, ENT, plastic and obstetrics and gynaecology operations, with 11,019 episodes of surgical care provided by 350 staff per year.

Participants were included if they were staff members working in the Sunshine Hospital operating theatre suite during the period 6^th^ November – 18^th^ December 2018. The craft groups included medical practitioners (anaesthetists, surgeons, obstetricians and gynaecologists, paediatricians and physician proceduralists and registrars in these specialties), nurses (anaesthetic, scrub/scout and paediatric nurses), midwives (attending theatre for deliveries) and theatre technicians. Participants were excluded if they were children, did not have a direct patient care role, were unable to provide written informed consent or had a known allergy to the fabric cap material.

The study operated in three phases. The first phase of the study consisted of recruitment and baseline surveying. During this time, the participants completed the pre-intervention survey after providing written informed consent. The specific questions are shown in Table 1. The participants also selected their preferred cap colour, name to be displayed and role descriptor.

**Table 1:** Survey Questions

1	Please tick your job role (from Nurse, Midwife, Doctor, Technician, Other)
2	Nurses only – what role did you work in for most of today (from Scrub / Scout Nurse, Anaesthetic Nurse, Recovery Nurse, Relieving Nurse, Admissions Nurse, DPU Nurse)
3	Doctors only – what role did you work in for most of today (from Anaesthetist, Anaesthetic registrar, Surgeon, Surgical registrar, Obstetrician and Gynaecologist, Obstetrics and Gynaecology registrar, Other proceduralist, Other proceduralist registrar)
4	Did you know the name of all of the anaesthetists (and registrars) you worked with today? (from Yes or No)
5	Did you know the name of all of the surgeons or obstetricians (and registrars) you worked with today? (from Yes or No)
6	Did you know the name of all of the nurses you worked with today? (from Yes or No)
7	Did you know the name of all of the technicians you worked with today? (from Yes or No)
8	Was there any point today where you couldn’t communicate with the team effectively about something? (from Yes or No)
9	How many staff in theatre in total did you NOT know the name of today?
10	Rate the teamwork you experienced in theatre on your shift today, from 0 – 5 (1 = completely unsatisfied, 2 = somewhat unsatisfied, 3 = neutral, 4 = somewhat satisfied, 5 = completely satisfied)
11	Did formal team introductions occur during time out? (from Yes, No and I’m not sure)
12	Which craft groups were present for the team introductions phase of the team time out during this list (tick all that apply) – from Scrub Nurses, Anaesthetic Nurses, Midwives, Surgeons, Obstetricians and Gynaecologists, or Other Proceduralists, Anaesthetists, Technicians, Paediatricians and team (if applicable) or None

In the second phase, enrolled staff were able to wear their cap in theatre for two weeks. This period was designed for the use of the caps to become standardized and for the effect, if any, of improved communication to be felt.

In the third phase participants were re-surveyed on their experiences using the same questions as in the first survey, referred to as the post-intervention survey. Participants were also posed an additional question of what impact they thought the caps had had on teamwork in the theatre complex.

**Fig. 1:**
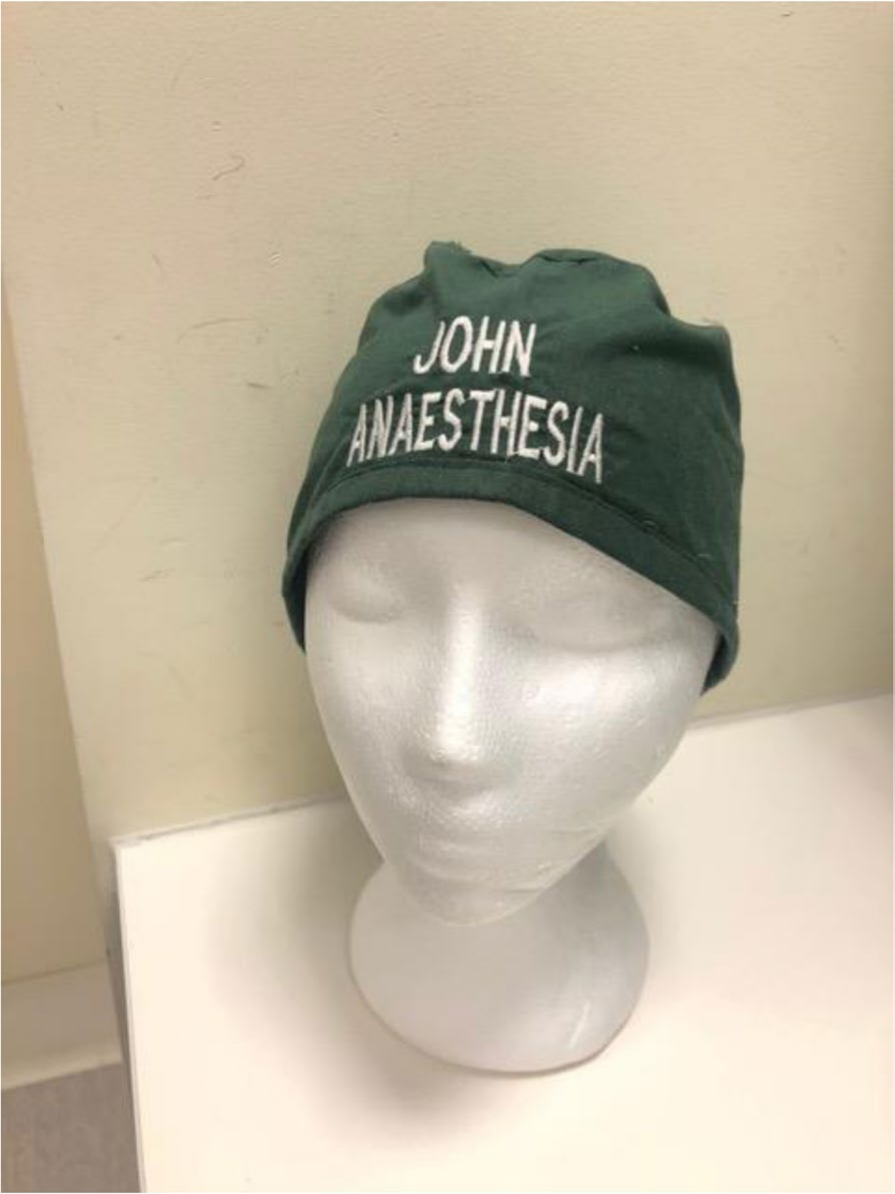
Typical cap

Fabric caps were ordered an online supplier of operating theatre clothing (rmfscrubs.com, Melbourne, Victoria, Australia). The text was centred in the middle of the cap and was 2.5 cm high and was embroidered using a high-contrast colour (for example, white text on a navy cap, black text on an orange cap). The name was on the top line and the role was on the bottom line. A typical example is shown in Fig. 1.

The study did not standardize cap colours for craft groups, nor did it standardize role descriptors. Participants could choose their own cap colour, name and role descriptor. Allowing customization felt to be important to encourage participation in the trial among the staff group and reflects real-world practice. A total of 35 non-patterned colours were available to choose from.

Staff were asked to wash the caps between each shift, in accordance with the guidance supplied by the Australian College of Operating Room Nurses. Staff were reminded to wash their cap at the start of each shift by the Associate Nurse Unit Manager of the operating suite. Staff who were unable to use their assigned cap could use disposable, non-named caps instead.

### Statistical analysis

Surveys were completed using an electronic form. Data was compiled in a spreadsheet (Microsoft Excel, Microsoft corporation), from which it was subsequently extracted and was analysed using SPSS v25 for Mac (IBM corporation).

The primary outcome was the perceived teamwork score rated using a 5-point Likert scale, where 1 represented completely unsatisfactory teamwork and 5 represented completely satisfactory teamwork. The median score was reported and the difference between the pre and post intervention scores was analysed using the Mann–Whitney U test. A sub-group analysis of the primary outcome in each of the craft groups (doctors, nurses, midwives and technicians) was pre-planned.

The number of unknown people in the theatre was reported as the median score and differences between pre and post intervention surveys were analysed using the Mann Whitney U test. The proportion of participants reporting they knew all of staff from each craft group was reported as a percentage and the difference between pre and post intervention proportions was analysed using a Chi-Squared test.

Statistical significance was pre-specified as *p* < 0.05 for the primary outcome. For the subsequent analyses, a Bonferroni correction was applied to correct for the total of ten secondary analyses, resulting in a new threshold of *p* < 0.005.

Qualitative data was analysed using immerse reading of the comments left by participants by one author (ND), from which themes were extracted.

## Results

A total of 236 participants were enrolled and completed the pre-intervention survey. The breakdown of participants by craft group on enrollment is shown in Table 2. A total of 107 participants subsequently completed the post-intervention survey. The reasons for loss of follow up were varied, and included a number of participants who had hats produced with their name or role printed upside down, a large number who had rotated or moved employment out of the health service (particularly midwives and registrars), and a number for whom the design of the hat was not suitable for their hair and subsequently did not participate in the trial.

**Table 2:** Participant enrolment by craft group

Craft Group	Number enrolled	Total working in theatre complex
Anaesthetist	37	60
Anaesthetic registrar	25	28
Nurse	48	110
Midwife	84	300
Technician	11	20
Obstetrics and Gynaecology Registrar	8	25
Other proceduralist Registrar	4	10
Surgeon	4	30
Surgical registrar	8	20
Obstetrician and Gynaecologist	4	30
Total	233	633

For the primary outcome, the median teamwork Likert scale score in the pre-intervention was 4 (IQR 3–5). This did not change in the post-intervention survey, where the median was also 4 (IQR 4 (IQR 2–5). Analysing the distribution of scores using the Mann–Whitney U test, the mean ranks were 163.81 in the pre-intervention survey, and 190 in the post-intervention survey, which was significantly different (U = 10 698.5, *p* = 0.015).

The results of the pre-planned subgroup analysis revealed the only group reporting a statistically significant improvement in perceived teamwork were midwives, who reported an increased median score from 3 to 4 (U = 757, *p* < 0.001). The distribution of scores were not significantly different for doctors (U = 2203, *p* = 0.899), nurses (U = 526.5, *p* = 0.73) or technicians (U = 33.9, *p* = 0.64), with all groups reporting a consistent median perceived teamwork score of four before and after the intervention.

The number of respondents who did not know the name of people in theatre also decreased after the intervention from a median of three pre-intervention to two post-intervention, which was significantly different (U = 8 657.5, *p* < 0.001).

The proportion of participants reporting knowing the names of all of the staff present in theatre more frequently after the intervention (31% vs 15%, χ^2^ = 20.2, *p* < 0.001). Participants reported knowing the name of the anaesthetists they worked with more frequently (81.3% vs 49.2%, χ^2^ = 31.5, *p* < 0.001). This change was not statistically significant for surgeons or obstetricians and gynaecologists (59.8% vs 47.9%, χ^2^ = 4.19, *p* = 0.47), or nurses (55.1% vs 43.6%, χ^2^ = 3.9, *p* = 0.062). Significantly fewer participants reported knowing all the names of the technicians they worked with in the post intervention survey (41% vs 59%, χ^2^ = 16.6, *p* < 0.001).

Formal introductions of the team as recommended by the WHO were reported to occur before the intervention by 34.7% of participants, with 53% reporting no introductions occurred in their theatre, and 12.3% being unsure. After the intervention the reported rates were 47.7% reporting introductions, 44.9% reporting no introductions and 7.5% being unsure. This change was not statistically significant (χ^2^ = 5.7, *p* = 0.058).

In the post-intervention survey participants could describe using free text the impact of the caps on theatre teamwork. Six common themes emerged in the participants’ experiences. The first theme concerned the use of the participants’ names in the clinical context. A typical example included a staff member who reported that another staff member, who they had worked with for years but had never used their name when addressing them previously, used their name on the first occasion they wore their intervention cap. The second theme concerned the effect of the hats on staff member’s willingness to communicate, which was perceived to improve when at least one staff member was wearing an intervention hat in theatre. The third theme referred to being able to address an unknown or unfamiliar staff member by name, which many participants reported was useful in facilitating communication with that person. The fourth theme described utility in being able to distinguish between functional roles in the team and address communication to the most relevant person. The fifth theme observed that more patients used staff members names to address them directly after the intervention was implemented. The last theme explained some of the struggles staff members had with the hats, specifically that the design used in the trial did not adequately cover many staff member’s hair. This particularly affected staff members with longer hair. An extension of this theme was dissatisfaction that only a single intervention hat had been provided, rather than sufficient hats to cover a week of work and allow efficient washing.

No adverse events to staff or patients were reported to the study team. One near-miss was reported, in that a participant’s hair fell from the cap whilst they were in theatre as the cap was not large enough to contain their long hair. This fall did not result in a compromise of sterility for the patient or instruments.

## Discussion

The introduction of theatre caps displaying staff members’ names and roles was associated with a significant increase in perceived teamwork among the staff of a general hospital operating theatre. Most of this increase is explained by the improvement in perceived teamwork by midwives attending the theatre for deliveries. This result fits with the hypothesis that the staff who are least often present in theatre are most likely to derive benefit from staff names being clearly displayed.

The fact that doctors, nurses and technicians did not perceive an improvement is likely due to three factors, first that the starting perception of teamwork was already high and secondly that those staff members worked together often and may have already known the majority of staff members names, limiting the impact of the intervention. The timing of the study, towards the end of the hospital employment year may have further reduced the impact of the intervention. Lastly, perceptions of teamwork may be influenced by many factors not addressed by the intervention such as tone and demeanor of staff, familiarity with tasks and clinical urgency and the impact of the intervention may have been insufficient to overcome these issues.

Whilst reported teamwork changed only in one group, the median number of unknown staff members was reduced for all participants, and this outcome is likely to be helpful to team performance. The broad increase in the number of staff knowing the names of the people they worked with is also reassuring. The fact that the largest and most significant increase occurred in anaesthetists likely reflected the high uptake of enrolment by this craft group.

The data support a role for theatre caps displaying a staff member’s name and role to become another tool in improving teamwork in theatre. The intervention is low cost, safe and appears to be effective.

The narrative analysis revealed important mechanisms by which the intervention exerted its effect, as well as a major problem in the delivery of the study. Organisations should consider customizing the fitting of hats to staff member’s individual needs and providing at least 4–5 hats when implementing this intervention.

The study experienced a number of limitations. Firstly, the inability to follow up all participants for the post-intervention survey introduces the possibility of selection bias. The authors feel this is less likely, as the majority of participants who declined to participate in the post-intervention survey did so because either they had not attended the operating theatre within the intervention period or had not worn their cap because it was not an appropriate fit for their hair. Secondly the fact that not all operating theatre staff were enrolled potentially diluted the impact of the intervention. Thirdly, a relatively low event rate for emergencies developing in the operating theatre during the study period limited the ability for participants to comment on the effectiveness of the intervention at the time that it would perhaps have the largest effect.

Future studies should address two questions left unresolved by this work, specifically whether the hats reduce the incidence of or improve the management of clinical crises, and secondly to evaluate the impact of the hats on patients’ experiences.

## Conclusion

The introduction of hats displaying the names and roles of staff members was associated with an improvement in perceived teamwork in an operating theatre of a general hospital. Improvements were seen most clearly in staff who attended theatre infrequently. Using the hats appeared to be safe. In deploying such hats, organisations should ensure the hats fit staff members appropriately. More precise research is required to address the contribution of such hats to crisis management and to evaluate their impact on patient-level outcomes.

## Data Availability

The datasets used and/or analysed during the current study are available from the corresponding author on reasonable request.
